# Traumatic Microhemorrhages Are Not Synonymous With Axonal Injury

**DOI:** 10.1002/acn3.70309

**Published:** 2026-01-20

**Authors:** Karinn Sytsma, Rhonda Mittenzwei, Heather Maioli, Amanda Kirkland, C. Dirk Keene, Ramon Diaz‐Arrastia, Christine Mac Donald, Chiara Maffei, Brian L. Edlow, Amber L. Nolan

**Affiliations:** ^1^ Department of Laboratory Medicine and Pathology University of Washington Seattle Washington USA; ^2^ Office of Chief Medical Examiner of the City of New York New York New York USA; ^3^ Department of Neurology University of Pennsylvania Philadelphia Pennsylvania USA; ^4^ Department of Neurological Surgery University of Washington Seattle Washington USA; ^5^ Athinoula A. Martinos Center for Biomedical Imaging, Department of Radiology Massachusetts General Hospital and Harvard Medical School Charlestown Massachusetts USA; ^6^ Center for Neurotechnology and Neurorecovery, Department of Neurology Massachusetts General Hospital and Harvard Medical School Boston Massachusetts USA

**Keywords:** axonal injury, neuropathology, traumatic microhemorrhages

## Abstract

Diffuse axonal injury (DAI) is caused by acceleration‐deceleration forces during trauma that shear white matter tracts. Susceptibility‐weighted MRI (SWI) identifies microbleeds that are considered the radiologic hallmark of DAI and are used in clinical prognostication. However, this assumption is limited by a lack of systematic radiologic‐pathologic correlation studies. Here, we performed ex vivo SWI on three brains from patients who died after severe TBI and assessed axonal injury around SWI microbleeds using immunohistochemistry to the amyloid‐beta precursor protein. Axonal injury was present in 64% of microbleeds, indicating a heterogeneous injury response in the white matter.

## 1 Introduction

Traumatic microbleeds are a hallmark finding in patients with diffuse axonal injury (DAI) [1, 2]. Rotational shearing forces that disrupt the microvasculature are likely to tear axons [3], such that the presence of a microbleed is interpreted as evidence of “hemorrhagic DAI” [4]. Accordingly, the presence of microbleeds factors heavily into clinical prognostication, because of the assumption that axons, and hence neural networks [5], are disrupted.

Yet emerging evidence has called the pathophysiologic link between traumatic microbleeds and DAI into question. When comparing vascular injury, as detected by susceptibility‐weighted imaging (SWI) or other magnetic resonance imaging (MRI) measures of vascular function, to the burden of DAI, as detected by diffusion MRI, several studies suggest that vascular injury and traumatic microbleeds may be dissociable from DAI [6, 7, 8]. In addition, two pathoradiologic studies of postmortem human tissue similarly suggest that axons may remain intact around traumatic microbleeds [9, 10]. However, systematic quantitative radiographic‐pathologic correlation has not been performed to examine this relationship.

Elucidating the pathophysiological relationship between microbleeds and DAI is not only of neuroscientific interest, but has immediate clinical relevance to patients with traumatic brain injury (TBI), for whom traumatic microbleeds are weighed heavily in prognostication. Indeed, the global burden of traumatic microbleeds [9], as well as the number of traumatic microbleeds in specific brain regions [11, 12], have shown associations with long‐term outcomes after TBI. Yet reports of functional recovery in patients with high numbers of global microbleeds [13], and multiple brainstem microbleeds [14], have reignited the debate about whether microbleeds are inextricably linked to debilitating DAI.

To elucidate the relationship between traumatic microbleeds and DAI, we performed ex vivo SWI on three brain donors who died less than 2 weeks after severe TBI. We used ex vivo SWI to guide histopathological sampling of white matter traumatic microbleeds and assessed axonal injury around these microbleeds using quantitative analysis of immunohistochemistry to the amyloid‐beta precursor protein (APP). Our observations weaken the pathophysiologic link between traumatic microbleeds and DAI.

## 2 Methods

**FIGURE 1:**
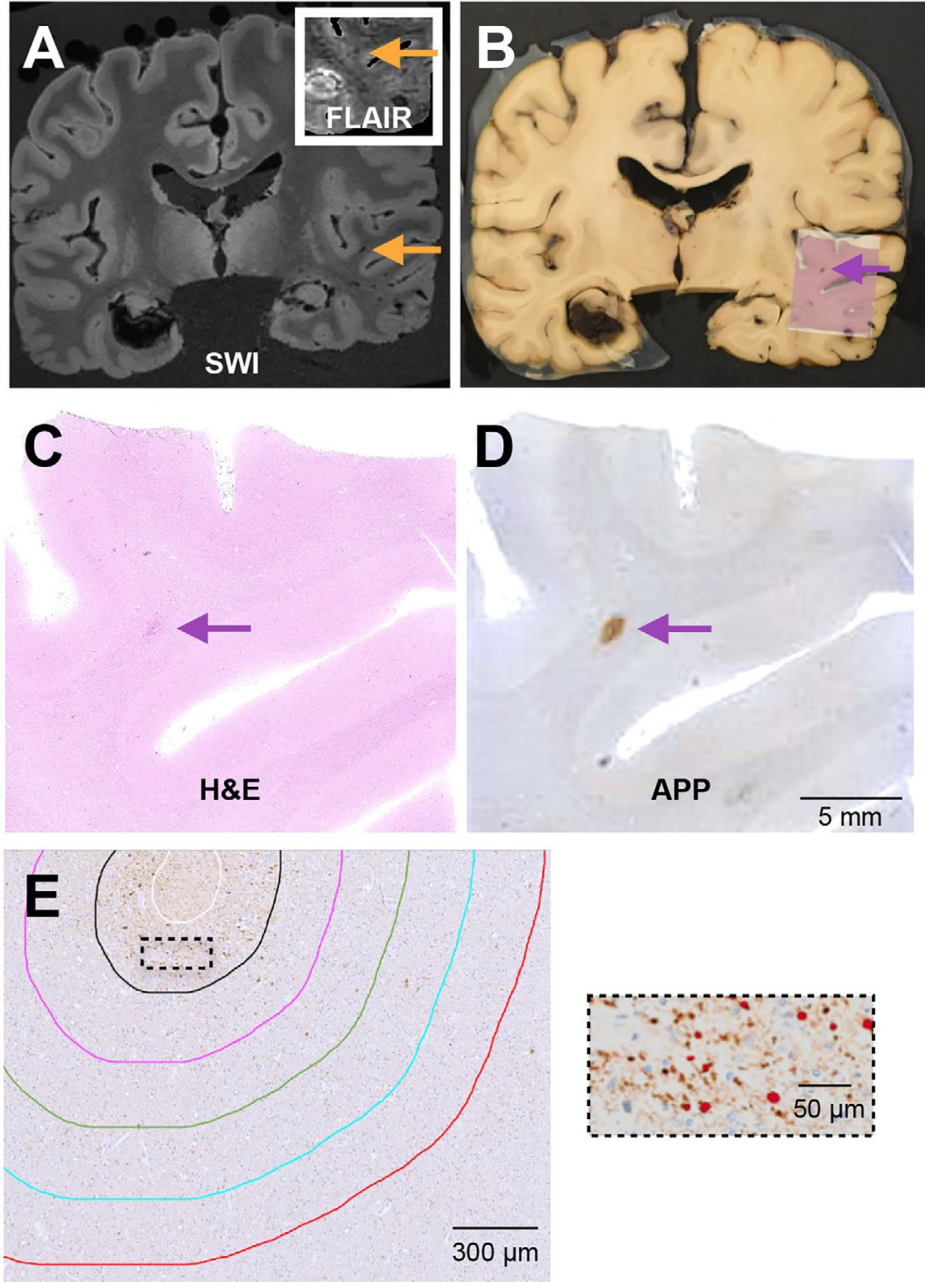
Methods to analyze axonal injury surrounding susceptibility weighted MR imaging (SWI)‐identified traumatic microbleeds. (A) Example coronal SWI image with traumatic microbleed denoted by orange arrow (and corresponding FLAIR image in the top right corner showing patchy increase in FLAIR hyperinsity around this lesion) with corresponding gross brain slice (B). The same traumatic microbleed is denoted by a purple arrow and an image of the hematoxylin and eosin (H&E) slide is overlying the sampled region. (C) The same H&E image from the sampled region and the sequential amyloid precursor protein immunohistochemical stained slide in (D). (E) Annotations for the regions of analysis performed in Halo. White line indicates the tracing around the microhemorrhage. Black, pink, green, blue and red lines represent 200‐μm concentric rings up to 1 mm from the microbleed. The dashed box is shown at higher power on the right with the Halo algorithm to identify axonal swellings shown in red.

In this case series, three brain donors (Table 1) with a history of severe TBI and death 1–2 weeks after injury were chosen for evaluation. Each donor underwent ex vivo MRI, followed by a neuropathologic evaluation as previously described [15]. To sample microbleeds, coronal brain slices of the cerebral hemispheres were spatially matched to images from the ex vivo SWI (Figure 1A,B). All regions with a white matter microbleed on SWI (defined as a punctate hypointense lesion at least 3 voxels in size located in at least 2 consecutive images at 500 μm resolution) were sampled (*n* = 34 blocks across 3 cases) and then stained with hematoxylin and eosin (H&E) and APP (Figure 1C,D). Of note, all SWI lesions also exhibited T2 FLAIR hyperintensity in adjacent white matter.

**TABLE 1:** Clinical features of brain donors.

Cohort	Case #1	Case #2	Case #3
Age	47	27	51
Gender identity	Male	Female	Male
Mechanism of injury	Pedestrian struck by vehicle	Fall from height (~3 stories)	Pedestrian struck by vehicle
Survival interval	7 days	13 days	12 days
Antemortem imaging	Diffuse subarachnoid hemorrhage, intraventricular hemorrhage, left frontal skull fracture	Sylvian fissure subarachnoid hemorrhage, grade 3 DAI	Right frontal contusion, grade 3 DAI
Past medical history	Unknown	History of alcohol use, depression, bipolar disorder	History of alcohol use

Abbreviation: DAI, diffuse axonal injury.

The presence of a microbleed was confirmed on H&E stain and only single microbleeds were included for analysis, given the focus on spatial analysis as a function of distance, and traced using the HALO platform (Indica Labs) (Figure 1E). Axonal injury was assessed within 200 μm concentric rings up to 1 mm from the microbleed by examining APP+ axonal swellings, which were defined as an object > 4 μm in diameter (Figure 1E). Although criteria have not been established for swellings that definitively represent an axonal transection, these values correspond to the size of axonal swellings that have been reported in the literature [16, 17]. For within‐case controls, the same concentric rings for each microbleed were placed randomly in the white matter on the same slide and examined.

Axonal injury was defined by the presence of > 3 swellings within one ring. To determine whether the relationship between microbleeds and axonal injury depends on neuroanatomic location, we classified each microbleed by lobe, location within the white matter (i.e., superficial or deep), and proximity to contusion. The presence of axonal spheroids on H&E stain was also determined for every microbleed. Selected slides with numerous microbleeds were also stained with neurofilament light (NFL) or neurofilament M (NFM) to compare to the APP results (See Appendix S1 for detailed methods).

Statistical analyses were performed using GraphPad Prism 9 software. The relationship of axonal swellings as a function of distance was analyzed with a Friedman test. A Fischer's exact test determined the association of APP+ axonal swellings with the presence of H&E axonal spheroids as well as neuroanatomic location and control sampling.

Unbiased clustering analysis of the density of APP+ axonal swellings was also performed to provide a more objective measure to classify microbleeds. A K‐means clustering algorithm, which optimizes the sum of the square Euclidean distance between points, was performed on the density of APP+ axonal swellings in the first 200 μm ring.

## 3 Results

**FIGURE 2:**
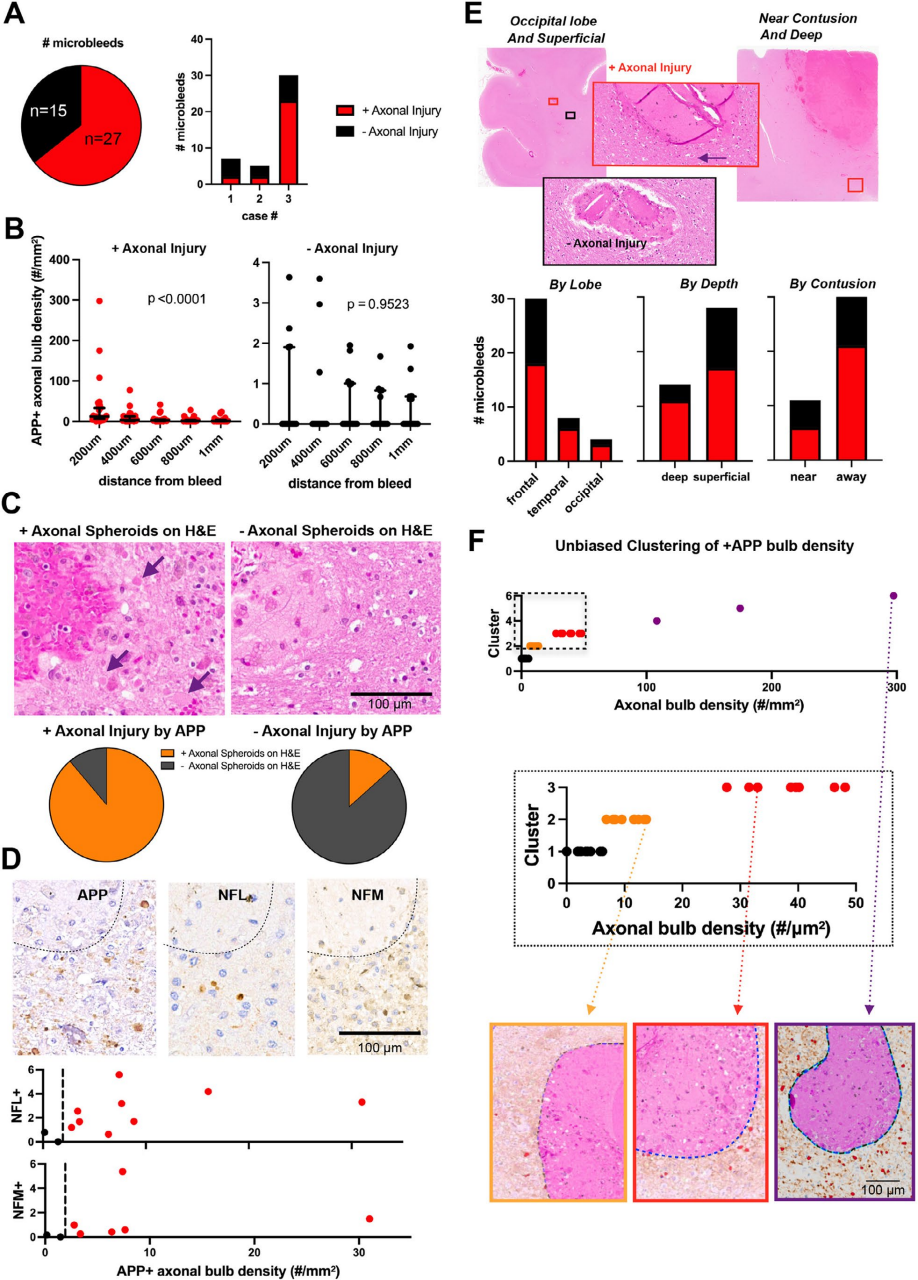
Axonal injury is not directly synonymous with traumatic microbleeds. (A) The number of microbleeds with axonal injury identified with amyloid precursor protein (APP) (27/42) and the microbleed distribution across cases. (B) The relationship between distance and APP+ axonal swellings in those considered positive for axonal injury on left (*p* < 0.0001, Friedman test) and without axonal injury on right (*p* = 0.9523, Friedman test). (C) Representative traumatic microbleeds with axonal spheroids (purple arrows) on hematoxylin and eosin (H&E) stain on the left and without axonal spheroids on the right. 24/27 traumatic microbleeds positive for axonal injury on APP had axonal spheroids seen on H&E. While only 2/15 traumatic microbleeds without axonal injury on APP stain exhibited an axonal spheroid on H&E. Fischer's exact test shows significant correlation (*p* < 0.0001) between axonal injury on APP stain and axonal spheroids on H&E. (D) Representative traumatic microbleed stained for APP, NFL and NFM (dotted line identifies hemorrhage) and plots showing correlation of NFL or NFM with APP axonal bulb densities. NFL and NFM show even fewer axonal swellings than the APP stain when correlating results for numerous microbleeds. Of note, the few negative microbleeds (black dots) analyzed did not show significant staining for either NFL or NFM. (E) Representative images of microbleeds in the occipital lobe and near a contusion. The red boxes indicate axonal damage on APP stain and H&E, the black box indicates no axonal injury. No significant relationship seen by location in lobe (*p* = 0.7737, Fischer's exact test), superficial versus deep (*p* = 0.3133, Fischer's exact test), or proximity to contusion (*p* = 0.4808, Fischer's exact test). (F) Unbiased clustering of the APP+ axonal swellings (bulbs) with the kmeans algorithm. There were three outliers with a very high density of APP+ swellings (purple), and then three other clusters with a minimal (black), mild (orange) and moderate (red) degree of injury.

Axonal injury was associated with 27/42 (64%) microbleeds, as defined by the presence of APP+ axonal swellings (Figure 2A). This proportion was substantially higher than that observed in the random analysis of white matter (3/42 randomly sampled regions, *p* < 0.0001, Fisher's exact test). The number of microbleeds and percentage with axonal injury was variable across cases (Figure 2A). The density of APP+ axonal swellings decreased as a function of distance for microbleeds positive for axonal injury (Figure 2B), and the presence of axonal spheroids on H&E was strongly correlated with the presence of axonal injury detected by APP stain (Figure 2C); similarly, NFL and NFM did not identify significant axonal injury in microbleeds negative for injury by APP (Figure 2D). The neuroanatomic classification of microbleeds did not affect the relationship between microbleeds and axonal injury (Figure 2E).

Unbiased clustering analysis of the density of APP+ axonal swellings revealed three outliers with a very high density of APP+ swellings (purple, Figure 2F), and three other clusters with a minimal (black), mild (orange), and moderate (red) degree of injury (Figure 2F). When this unbiased tool is used to define axonal injury, the minimal group that might be inferred to represent microbleeds without significant axonal injury included 23 microbleeds (55%), raising even higher the number of microbleeds “negative” for axonal injury.

## 4 Discussion

We performed systematic quantitative radiologic‐pathologic analysis to determine the relationship of axonal injury to traumatic microbleeds identified on SWI in three brain specimens donated by patients with acute severe TBI. We found that a subset (64%) of microbleeds exhibit adjacent axonal injury, supporting a probabilistic association rather than a 1:1 direct relationship. Our data also reveal a variable severity of axonal injury, as the majority of microbleeds with adjacent axonal injury had few APP+ axonal swellings.

While this is the first quantitative analysis of microbleeds and axonal injury, our data align with prior observations. In a patient who died 7 months after severe TBI, microbleeds on postmortem MRI were found to exhibit iron deposition and hemosiderin‐laden macrophages without axonal beading or injury [9]. In a patient who died 3 days after injury, postmortem MRI and pathological anlaysis revealed a cingulum bundle hemorrhage without associated APP+ axons [10]. In a swine model of rotational acceleration, inverstigators observed fibrinogen extravasation, a marker of blood–brain barrier disruption, in the absence of axonal injury [18], consistent with our observations that vascular and axonal injury are not inextricably linked.

Why some microbleeds are associated with axonal injury is unclear. We examined the location of microbleeds by superficial versus deep white matter, lobe and proximity to contusion, and none of these metrics were associated with the presence of axonal injury. An imaging study suggested that microbleeds in the midsagittal region (cingular cortex, parasagittal white matter, and corpus callosum) are more likely to be associated with axonal injury [6], but most microbleeds in this study were in lobar regions of the brain [11, 12]. The specific white matter pathways that a vessel is associated with might influence the likelihood of axonal injury, with long‐ranging pathways being perhaps more vulnerable. This hypothesis will require future testing with ex vivo diffusion MRI tractography. Cellular responses to the microbleed could also variably affect axonal integrity due to the glial heterogeneity that has been found across regions of the brain [19].

Our study has several limitations. These patients died 1–2 weeks after injury, raising the possibility that pathology might be secondary to other processes such as ischemia and swelling that occur after severe TBI. We also only analyzed “single” microbleeds away from other microbleeds. Children, who might exhibit a stronger relationship between SWI microbleeds and axonal injury [20], were not examined in this study. While APP, NFL, and NFM do identify a majority of axonal injury, there is a subset of damaged axons that do not stain with these markers [17] and we cannot entirely exclude that microbleeds negative for injury might show pathology with other markers; although it should be noted that H&E assessment also correlated very well with our APP results. Finally, the ex vivo SWI and subsequent sampling did not include the brainstem, which is routinely removed before imaging in our protocol and an area of great prognostic relevance, especially regarding recovery of consciousness [10]. Future studies will need to incorporate more cases with variable severity, timing, and age, a greater number of regions including the brainstem, and clusters of microbleeds to determine if the principles observed here are generalizable.

In summary, our systematic quantitative evaluation of microbleeds weakens the dogma of “hemorrhagic diffuse axonal injury.” These results provide the basis for a reevaluation of prognostic models in TBI that strongly rely upon traumatic microbleeds identified on imaging.

## Author Contributions

C.D.K., R.D.‐A., and B.L.E. were involved in the brain donation of these cases. R.M., H.M., C.M., and C.M.D. identified the microhemorrhages on imaging and sampled the brain tissue. A.K. performed the histology and staining. R.M., H.M., K.S., and A.L.N. performed the Halo annotations and analysis. B.L.E., R.D.‐A., C.M.D., C.M., and A.L.N. contributed to the design and approach of the study. A.L.N. and B.L.E. wrote the paper.

## Funding

This work was supported by NIH/NINDS (K08NS114170, R01NS138257, R21NS109627, R01NS091618, U01NS086090, U54NS115322, U01NS137500, U24NS135561, RF1NS115268, U01NS086625, U01NS137484), NIH/NIA (P30AG066509, AG066509, U24AG072458), NIH Director's Office (DP2HD101400), the United States Department of Defense (W81XWH‐21‐S‐TBIPH2), the VA Advanced Fellowship in Mental Illness Research and Treatment, the Chen Institute MGH Research Scholar Award, and the Nancy and Buster Alvord Endowment.

## Conflicts of Interest

The authors declare no conflicts of interest.

## Supporting information


**Appendix S1:** acn370309‐sup‐0001‐AppendixS1.docx.

## Data Availability

The data/images used and/or analyzed during the current study are available from the corresponding author on reasonable request.
