# Supplementary material for: Traumatic Microhemorrhages Are Not Synonymous With Axonal Injury
Source: Ann Clin Transl Neurol. 2026 Jan 20;13(5):1060–5. doi: 10.1002/acn3.70309 (PMC13161866; doi:10.1002/acn3.70309)
Supplement: Supplementary file 1 — Appendix S1: acn370309‐sup‐0001‐AppendixS1.docx. [file ACN3-13-1060-s001.docx]

**SUPPLEMENTARY MATERIAL**

**METHODS IN MORE DETAIL**

The three cases for evaluation from the University of Washington Neuropathology laboratory included two from the Ex Vivo Connectomics study at Massachusetts General Hospital and one from the Brain Oxygen Optimization in Severe TBI Phase-3 (BOOST3)/Transforming Research and Clinical Knowledge in Traumatic Brain Injury (TRACK-TBI) study at the University of Pennsylvania. All studies were approved by the Massachusetts General Hospital, University of Pennsylvania, and University of Washington Institutional Review Boards and/or School of Medicine Compliance offices. Informed consent for brain donation was provided by legal next of kin. All cases underwent *ex vivo* MRI, followed by a neuropathologic evaluation as previously described.^1^

After sampling blocks for microbleeds, hematoxylin and eosin (H&E), APP, neurofilament light (NFL), and neurofilament M (NFM) staining was performed on a Biocare autostainer (APP: MAB348 Sigma-Aldrich, 1:75 dilution, epitope retrieval time 15 min; NFL: -1391-50 Biosensis, 1:200 dilution, epitope retrieval time 8 min). Slides were scanned with an Aperio AT2 Leica slide scanner at 20x. Image analysis was performed in HALO image analysis software (Indica Labs). The presence of a microbleed was confirmed on H&E stain based on the presence of red blood cells in the parenchyma. Only single microbleeds were analyzed defined as >1.5mm away from another microbleed. In addition, only microbleeds >500μm in diameter were included to correlate with a size could be identified on imaging. 200μm concentric rings up to 1mm from the microbleed were placed around every microbleed on the APP-, NFL- and NFM-stained slides. An axonal swelling was defined as an object >4μm in diameter using a pre-determined intensity threshold (found by adjusting the minimum optical density of the stain to be considered positive while also avoiding background stain). Developed analysis settings were checked with multiple images for each stain, and batch analyzed using the same parameters across images. Axonal injury was defined by the presence of >3 swellings within one ring; this threshold was chosen to reduce the false positive rate associated with the algorithm, rarely identifying inappropriate background staining. The same annotations around analyzed microbleeds were placed randomly in the white matter on the APP-stained sections and examined. Microbleeds were excluded from analysis if too much background staining was present and extensively interfered with the Halo imaging algorithm, as visually inspected by a neuropathologist. Several slides that contained multiple microbleeds were stained with NFL and NFM. A similar algorithm was used for analysis, but given increased formalin pigment deposition and background staining interfering with the image analysis algorithm, the NFL+ and NFM+ bulb counts were manually confirmed by a neuropathologist. The presence of axonal spheroids on H&E stain was also determined for every microbleed by a neuropathologist, blinded to the results of the APP analysis. An axonal spheroid was defined as a granular eosinophilic abnormal round swelling.

**REFERENCES**

1. Latimer CS, Melief EJ, Ariza-Torres J, et al. Protocol for the Systematic Fixation, Circuit-Based Sampling, and Qualitative and Quantitative Neuropathological Analysis of Human Brain Tissue. Methods Mol Biol. 2023;2561:3-30.
